# WASH policy landscape: exploring context, process, content, and actors in childcare centres in Nairobi’s informal settlements

**DOI:** 10.1186/s12889-026-28653-x

**Published:** 2026-07-24

**Authors:** Ivy Chumo, Caroline Kabaria, Helen Elsey, Penelope A Phillips-Howard, Blessing Mberu

**Affiliations:** 1 African Population and Health Research Center (APHRC), Nairobi, Kenya; 2 University of York, Hull York Medical School, York, UK; 3 Department of Clinical Sciences, Liverpool, 3 Liverpool School of Tropical Medicine, Liverpool, UK; 4 Demography and Population Studies, University of Witwatersrand, Johannesburg, South Africa

**Keywords:** WASH, Policy review, Informal settlements, Childcare centres, Kenya

## Abstract

**Introduction:**

Adequate water, sanitation, and hygiene (WASH) services are fundamental to health and development. However, many underserved areas such as informal settlements lack these services, creating significant infrastructural and public health challenges. Inadequate WASH infrastructure in Nairobi’s informal settlements reveal significant gaps in childcare centre guidelines. Integrating robust guidelines and quality WASH services into childcare centres optimize operations and directly improves children’s wellbeing. This study explores the policy environment governing WASH service delivery in these settings. We analysed the policy landscape’s content, context, actors, and processes.

**Methods:**

This study employed a dual-methodological approach within Nairobi’s informal settlements. First, we systematically reviewed 25 policy documents to analyse the content, context, actors, and processes governing WASH guidelines, utilizing Walt and Gilson’s Policy Triangle Framework. Second, we conducted 32 in-depth interviews (IDIs) and 8 key informant interviews (KIIs) with policymakers, parents, and childcare providers. These interviews used the same framework to identify policy gaps and practical implementation barriers.

**Results:**

Results revealed a systemic fragmentation within the WASH policy landscape. While water (88%) and sanitation (80%) guidelines appeared frequently, hygiene guidelines were significantly less represented (68%). Overlapping mandates among the Ministries of Health, Education, and Water and Sanitation hindered effective implementation of policy guidelines. Gaps in the policy process and fragmented inter-sectoral coordination weakened the overall governance of WASH services and practices. This lack of institutional synergy ultimately resulted in suboptimal and precarious WASH outcomes within informal settlements.

**Conclusions:**

Current WASH policy frameworks are misaligned with the realities of informal urban settlements. Existing documents have inadequate strategies for these environments and for children under five. This contextual disconnect, coupled with the barriers of persistent poverty, means that service delivery often remains stalled or unsustainable. Bridging the gap between policy and practice requires moving beyond the policy content to the “how” of the policy process. Relevant actors in the sector should move towards a governance model that prioritizes a cohesive, politically supported, and monitored architecture for tangible community impact.

**Supplementary Information:**

The online version contains supplementary material available at https://doi.org/10.1186/s12889-026-28653-x.

## Introduction

Adequate water, sanitation and hygiene (WASH) services are essential for child health and wellbeing [1]. These services play a central role in realizing children’s rights [2, 3]. Globally, the COVID-19 pandemic revealed the importance of safely managed WASH services and practices for controlling the spread of infectious diseases, sustainable community livelihoods and wellbeing [4, 5]. The rapid spread of the pandemic called for large investments in healthcare systems, WASH policies, and infrastructure [6]. Although access to WASH services establishes lifelong hygiene practices, inadequate access compromises health and wellbeing [7, 8]. It further creates significant physical barriers in childcare centres, increasing the risk of infection from unsafe facilities [1, 8]. To guarantee the right to WASH services, centres should meet specific regulatory and infrastructural requirements [8]. Effective WASH interventions in childcare centres can significantly reduce absenteeism caused by disease and other developmental challenges [1, 9].

More than 250 million children globally do not reach their developmental potential due to early childhood adversities [5]. In Africa, accelerating and unplanned urbanization forces an increasing number of children into informal settlements [10]. In Kenya, childcare centres, which primary serve children aged below 5 years [9, 10], prepare the children for academic success and healthy development [11, 12]. These institutions are variously referred to as Early Childhood Development and Education (ECDE) centres, pre-schools, pre-primary or day-care centres [11, 13]. Childcare centres are a subset of ECDE services in the Kenyan context. Quality service delivery in Kenya relies on pre-primary policy guidelines [14]. Sustainable WASH services and practices within childcare centre programs require leadership from the Ministry of Education as well as related Ministries such as Health, Social Services, Finance, Local Governance, Sanitation and Water Authorities [15]. Historically, this sector involves many stakeholders ranging from parents, religious organizations, Non-Governmental Organizations (NGOs), private sector/individual, and different sectors in the government. Childcare centres in Kenya report to different departments within the county governments, and there is a need to track performance progress in service delivery for the counties [12, 16]. This underscores the need to understand policy guidelines for WASH services in Kenya and particularly for children [12].

Nairobi typifies Africa’s rapid urbanization and growth of informal settlements, with approximately 60% of the city’s population now living in informal settlements [17, 18]. Changing social and family structures with more working mothers, single parents, nuclear families and older siblings enrolled in schools have increased the demand for childcare services [9, 18]. Childcare centres have a growing significance in shaping life course trajectories for children, yet many centres in informal settlements do not have adequate WASH services [12, 16]. This gap has raised critical concerns regarding the adequacy of WASH service delivery within these settings [19–23]. While previous policy reviews have predominantly focused on narrow content analysis [22, 24], however, an urgent need now entails mapping the broader policy ecosystem in informal settlements [2, 8, 16] to incorporate context, process and actors in childcare centres.

The study explored the WASH policy landscape in childcare centres, focusing on those in Nairobi’s informal settlements. We aimed to fill a literature gap by examining policy context, process, content, and actors. In addition to a secondary policy review, we used primary qualitative data to show how policy gaps affect actual service delivery.

## Methods

This study utilized a two-pronged approach guided by Walt and Gilson’s (1994) policy triangle framework [25]. This framework provided the methodological foundation for examining policy content, context, process, and actors. We first reviewed 25 existing policies to identify relevant WASH guidelines. Following this analysis, we collected primary qualitative data through 8 key informant interviews (KIIs) with policymakers and 32 in-depth interviews (IDIs) with parents and childcare providers. This combined approach allowed us to identify policy gaps and implementation barriers using Nairobi County as a case study, filling a significant knowledge gap regarding childcare centres in informal settlements.

### Conceptual framework

**Fig. 1:**
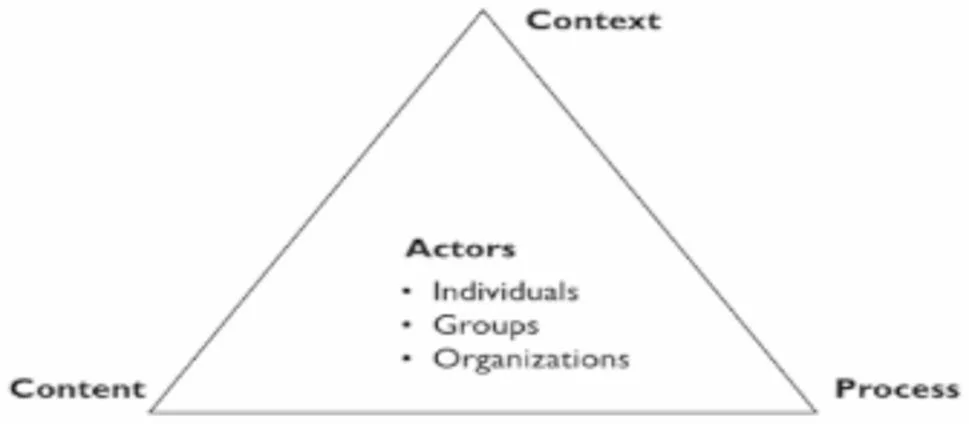
Walt and Gilson’s policy analysis framework [26]

The Policy Triangle Framework developed by Walt and Gilson [25] informed the study (Fig. 1). This framework offers a comprehensive model for policy analysis by emphasizing that comprehensive understanding of a policy landscape requires looking beyond its content to include other features. It highlights the importance of considering the policy’s actors, process and context. The policy triangle considers how these aspects of policy interact and shape policy and practice in relation to content, context, process and actors in the selected policies [26].

The Policy Triangle consists of four elements:

1. Actors: individuals, organizations or the state and their actions that affect policy implementation. Who participates in and influences formulation and implementation of the policy? Are specific roles for different actors described?
2. Context: Local, national or international contexts which may affect the policy. Where do they need the policies?
3. Content: Substance of the policy. What is the policy mainly about?
4. Process: The way policies are developed and implemented. How was the policy brought forward and implemented?

### Policy review and analysis

#### Search strategy

Our search strategy targeted various sectors, including Water, Sanitation, Hygiene, Education, Health, Labour, Finance, and Social and Child Protection. We specifically aimed to identify policies within the corresponding sectors of the Kenyan government. Given that early childhood matters are handled at the county level in Kenya, we only included the Nairobi County Integrated Development Plan (CIDP). Consequently, guidelines from other counties are not applicable in early childhood, as each county has its own specific guidelines.

**Fig. 2:**
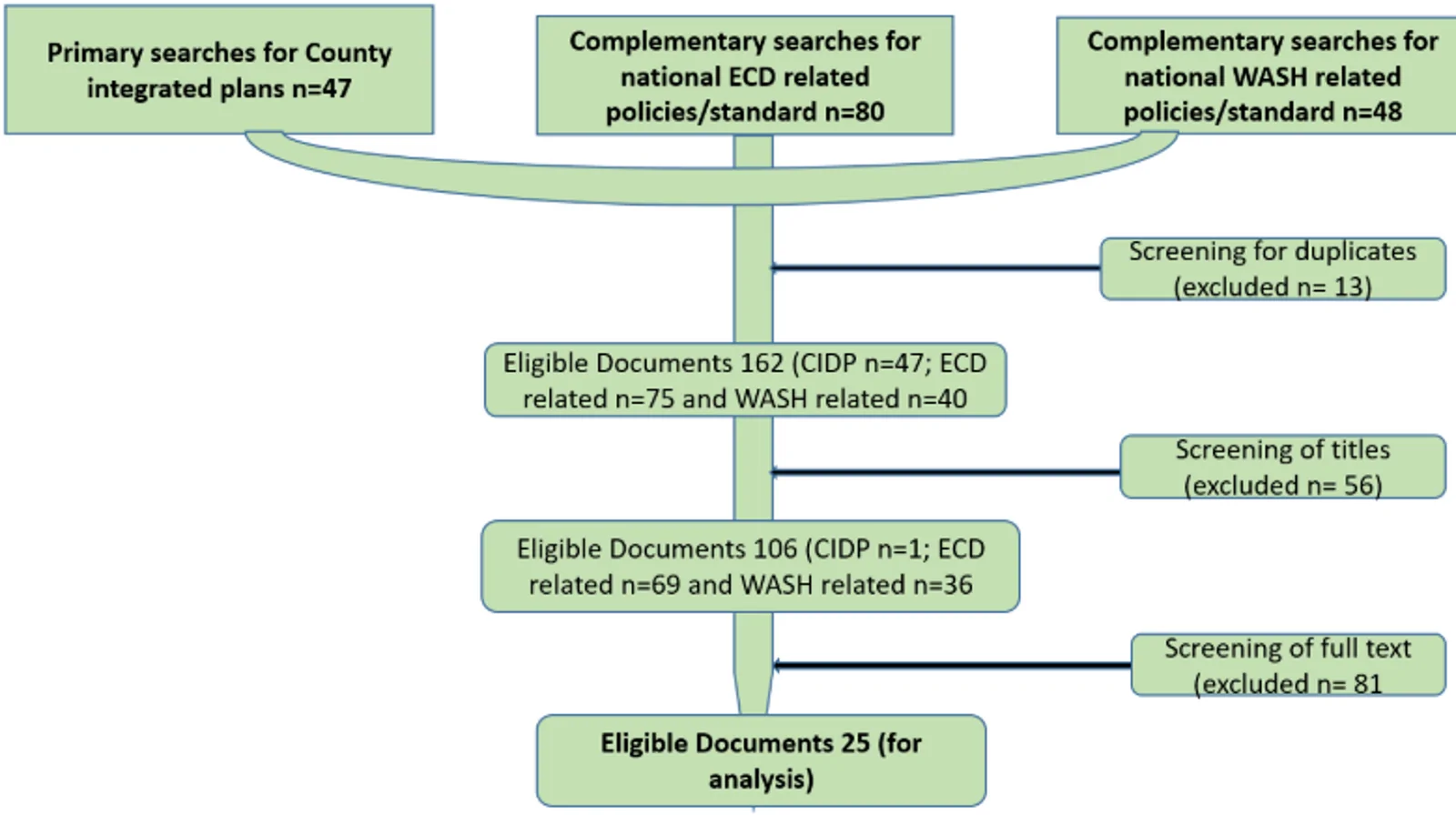
Screening process

To retrieve policy documents, we conducted a primary search in the e-repository for Kenyan government documents, government ministries (water, sanitation and irrigation; health; education; children, and social services; labour, and social security and services) websites, Nairobi County website and national councils’ websites among others. The review process included strategy implementation plans, policy frameworks and plans. The methodology was earlier documented in “*Improving early childhood development in the context of the nurturing care framework in Kenya: A policy review and qualitative exploration of emerging issues with policy makers*” [27] and in the “Development of the National Sanitation Management Policy (NSMP) in Kenya” [28]. Search terms included keywords such as “WASH” and “early childhood development,” among others outlined in Table 1. We proceeded to drop duplicates; including those with an eligible title but ineligible content, and to exclude documents where full text was not available (Fig. 2). We also excluded documents with policies authored before 2011, at the inception of a new constitution in Kenya. The information gathered from secondary sources was organized, summarized and categorized according to themes and objectives of the review. Walt and Gilson (1994) policy triangle ensured the analysis follows the “content, context, process, and actors” structure. The information was collated and triangulated into comprehensive findings.

**Table 1:** Search terms for policy review

Search terms
Early childhood development
Policies & guidelines
Centre-based care
Children under-5
Community-based care
WASH
Water
Sanitation
Hygiene
Informal settlements
Urban areas
Nurturing care framework
CIDP-Nairobi County
Stakeholders
Multilateral organisations/donors (Kenya specific policies)
Policy implementation strategies
Policy Implementation Frameworks / Plans

Inclusion Criteria: Policies mentioning any of the search terms in Table 1;

Exclusion Criteria: Policies documented before 2011 (at the inception of Kenya’s new constitution).

Out of the initial 175 retrieved documents, duplicates were removed (*n* = 13), titles were screened out (*n* = 56), and full-text screening excluded 81 documents based on strict criteria (e.g., documents authored before the 2011 constitution inception were excluded). The text from the 25 selected documents was extracted and systematically coded into an Excel matrix matching the four quadrants of the Walt and Gilson framework (Content, Context, Process, and Actors) to ensure qualitative transparency and reproducibility.

### Qualitative data collection and analysis

#### Study setting

Qualitative data were collected to complement the policy review data. Primary data collection was conducted in Korogocho and Viwandani informal settlements. These settlements are within the Nairobi Urban Health and Demographic Surveillance System (NUHDSS), initiated in 2002 by the African Population and Health Research Centre (APHRC) [29]. Korogocho features a stable, settled population [30], while Viwandani is located near an industrial area, and has highly mobile and educated residents [30].

Previous studies have documented the WASH service delivery in Korogocho and Viwandani as a mix of both formal and informal actors [31, 32]. The studies further explain that childcare centres rely on a mix of WASH services like boreholes, portable water and sanitation facilities and pit latrines [23, 33]. The service providers in this context are a mix of government officials and informal actors, such as community-based organizations and private vendors, who manage and maintain these services [31, 34].

#### Target population, sampling and sample size

The population of interest were policy actors, parents and childcare providers. From the stakeholder mapping exercise, we developed a database of actors and service providers. This database was later confirmed by the study participants during the governance diaries data collection process [34] and community advisory study consultation [35]. We collected qualitative data through 8 KIIs and 32 IDI eight KIIs and thirty-two IDIs across both study sites. KIIs were conducted with staff from the Nairobi County Government Ministries of Education (*n* = 2), Water & Sanitation and Irrigation (*n* = 2), Gender and Social Development (*n* = 2), and Health (*n* = 2). These are the key ministries responsible for WASH services in childcare centres as depicted while mapping actors and networks in WASH service provision [31]. The ministry staff were purposively selected based on their specific roles in WASH/Education departments. The 32 IDIs consisted of one-on-one conversations with 8 parents and 8 childcare providers in each study site who were mostly involved in the day-to-day activities. The criteria for the selection of childcare providers were based on those that had been in existence for more than 8 years to ensure that they had experience with policy shifts. In each of the centres where a childcare provider was purposively recruited, one parent was also purposively selected. Data collection continued until thematic saturation was achieved at the fourth participant for both cohorts within each settlement, as no new insights emerged. We purposively selected parents/guardians who were responsible for household child-rearing expenses in the family. The selection criteria enabled us to engage participants with long tenure in the study sites and with experience in WASH service delivery.

#### Data collection process

We collected data from March to May 2022 using an interview guide. We selected research assistants who had some experience in qualitative research and were endorsed by the local community advisory committees [35]. Research assistants from the community bring contextual, embedded knowledge, which outsiders may not be able to uncover [36]. Research assistants were trained for five days on study aims, data collection procedures, study tools, and study ethics by the ARISE Consortium project team. The interviews were conducted face-to-face in English or Kiswahili for approximately one hour at the participants’ workplaces or community spaces. Data was collected using digital recorders and supplemented with handwritten summary notes. To ensure data quality, researchers reviewed audio recordings in real-time, provided feedback to assistants, and conducted daily debriefing sessions to identify emerging themes, refine interview techniques, and monitor overall project progress.

#### Data management and analysis

We transcribed audio recordings verbatim and translated them from Kiswahili to English as needed. Each transcript was saved as an individual Microsoft Word document, assigned a unique identification code, and imported into NVivo 12 software (QSR International, Australia) for qualitative analysis. To maintain confidentiality, transcripts were anonymized using codes that indicated, participant category, study site, and sex.

We used a framework analysis [37], guided by the policy analysis triangle [24]. This method is suitable for research with specific questions and pre-designed sample and priory issues [37]. Researchers initially reviewed the transcripts to become familiar with the data and overall context. To ensure reliability, a coding framework was developed collaboratively by two experienced qualitative researchers and two data-collecting research assistants. This process included independent coding of transcripts in NVivo 12 to establish inter-coder agreement. Following the development of the initial coding framework, the research team met to discuss and refine themes and subthemes (Table 2). Subsequently, the two researchers conducted in-depth coding, charting, and mapping of transcripts to interpret the data.

#### Ethical considerations

The AMREF Health Africa Ethics & Scientific Review Committee (ESRC) approved this study (AMREF-ESRC P747/2020). Approval letters were also obtained from all the relevant authorities at Liverpool School of Tropical Medicine (LSTM), African Population and Health Research Center (APHRC), National Commission for Science Technology and Innovation (NACOSTI) and at the community level before commencement of study activities. Research assistants read the information sheet to study participants before the interview.

Informed consent was obtained from all participants, explicitly covering both their agreement to participate and their permission for the sessions to be audio-recorded. To ensure ethical participation, we provided a comprehensive Informed Consent Form (ICF) to every participant. This form explained the study’s objectives, the interview process, and how we would use the data. It also emphasized the right to withdraw at any time, identified our institutional affiliations, and outlined confidentiality protocols. The interviewer explained the contents of the information sheet to the participants and gave them an opportunity to ask any questions. Participants provided signed affirmation on the ICF confirming their voluntary acceptance of both participation and the audio recording. At the start of every recorded session, the researchers verbally confirmed and captured the participant’s final agreement on the recording, creating a documented record of consent for all collected data.

## Results

In this section, we present the results of our policy landscape document analysis and report qualitative data findings to complement this secondary analysis.

Our search identified a total of 175 policy documents including 47 County Integrated Development Plans (CIDPs), 80 national ECD/ childcare centre policy documents and 48 national WASH policy documents (Fig. 2).

We included 25 full text documents in final synthesis Table 2.

**Table 2:** Policy analysis results in context to Early Childhood Development (0 = No; 1 = Yes;2 = Unclear)

Document Title	Policy Context			Policy Content						Policy Process	Policy Actors	Summary
Policy details				Centre-based care	**Urban/informal settlements**		**WASH in childcare centres**			**Process Actors**	**Implementation Actors**	**Policy Status**
Document title	**Year published**	**Policy focus: National/ County**	**Age group(s) 0 to 5 years**	**Does the policy mention centre-based?**	**Does the policy mention urban areas?**	**Does the policy mention informal settlements?**	**Does the policy mention about Water?**	**Does the policy mention about Sanitation?**	**Does the policy mention about Hygiene?**	**Were there both childcare centres and WASH actors in development?**	**Are there specific roles designed for stakeholder in implementation?**	**Policies that meet requirements (Frequency (%)**
Nairobi County Integrated Development Plan 2018–2022	2018	County	1	1	1	1	1	1	1	1	1	9 (100%)
The framework for the national child protection system.	2011	National	3	0	0	0	1	1	0	1	1	4 (44%)
The National Plan of Kenya against sexual exploitation of children	2013	National	1	0	0	0	0	0	0	1	1	3 (33%)
Policy for management of diarrhoea in children below 5	2014	National	1	3	1	0	0	0	0	3	3	2 (22%)
National framework and plan of action for implementation of integrated community case management (iCCM) in Kenya	2013	National	1	3	1	0	1	1	1	0	3	5 (56%)
Laws of Kenya, Children Act, Chap. 141	2012	National	1	0	0	0	1	0	0	0	1	3 (33%)
Kenya National Nutrition Action Plan	2018	National	1	1	1	0	1	1	1	1	1	8 (89%)
National Plan of Action for Children in Kenya	2015	National	1	1	1	1	1	1	1	3	1	8 (89%)
National Standards for Best Practice in Charitable Children’s Institutions	2013	National	3	0	0	0	1	1	1	1	1	5 (56%)
Kenya national social protection policy	2011	National	3	0	1	1	0	0	0	3	1	3 (33%)
National School Health Strategy Implementation Plan	2011	National	3	0	3	0	1	1	1	1	1	5 (56%)
Guidelines for the alternative family care of children	2014	National	1	1	1	1	1	1	1	1	3	8(89%)
Kenya reproductive, maternal, newborn, child and adolescent health (RMNCAH)) investment framework	2016	National	1	0	1	1	1	1	1	1	1	8(89%)
National pre-primary education policy	2017	National	1	1	1	1	1	1	1	1	1	9 (100%)
Footprints children’s home child protection policy	2017	National	3	0	0	0	1	0	0	3	3	1 (11%)
The National Children’s Policy	2010	National	3	0	0	0	1	1	1	3	3	3 (33%)
Policy for Learners and Trainees with Disabilities	2018	National	3	0	1	1	1	1	1	3	1	6 (67%)
Kenya Health Policy	2012	National	3	0	1	1	1	1	0	1	1	6 (67%)
National Maternal, Infant and Young Child Nutrition policy guideline	2013	National	3	0	1	1	1	1	1	3	1	6 (67%)
National Pre-primary education policy standard guidelines	2018	National	1	1	3	0	1	1	1	1	1	7 (78%)
National Environmental Sanitation and Hygiene Strategy (2010–2015)	2011	National	3	3	0	0	1	1	1	1	1	5 (56%)
The Kenya Vision 2030	2012	National	3	0	1	0	1	1	0	1	1	5 (56%)
The Environmental Health and Sanitation Bill	2020	National	3	3	1	0	1	1	1	1	1	6 (67%)
Kenya Environmental Sanitation and Hygiene Policy	2016	National	1	1	1	1	1	1	1	1	1	9 (100%)
Policy Framework for Education	2012	National	1	1	1	1	1	1	1	1	1	9 (100%)
Frequency of policy (Yes)			**13**	**8**	**16**	**11**	**22**	**20**	**17**	**16**	**20**	**143/225**
Percentage of policy (Yes)			**52**	**32**	**64**	**44**	**88**	**80**	**68**	**64**	**80**	**63.5%**
% of policy content, context and actors (Yes) with age group below 5 & centre based & informal settlements			**-**	**-**	**-**	**-**	**100%**	**100%**	**100%**	**83%**	**83%**	

### Policy content

We reviewed 25 policies and identified significant content gaps across the three WASH pillars. Most policies included at least one pillar, but coverage remained inconsistent. Water guidelines appeared in 22 policies (88%), while sanitation appeared in 20 (80%). Hygiene guidelines lagged, appearing in only 17 (68%) of the reviewed documents. Although policies shared similar objectives, they differed in how these were defined. In most cases, hygiene was embedded within the broader health or education policies which reduced its visibility and limited its impact within the sector.

Findings from the primary data collection validated the policy review. Participants highlighted the need for integrated policies that guarantee equitable access to water, sanitation, and hygiene for all. A policy actor noted that the presence of “clear guidelines” does not automatically translate into high-quality infrastructure. Study participants described how childcare centre owners demonstrated a profound lack of mindfulness regarding mandatory WASH standards. The participant further explained how this negligence created substandard conditions that directly undermine the safety of the children in their care.

*“We need policies and frameworks that address both water*,* sanitation and hygiene for all. Sometimes there are no clear guidelines for WASH in many policies…some early childhood centre owners are not mindful of the guidelines. You will find that the WASH amenities are pathetic”* (Policy Actor, KII Nairobi).

We also explored the Policies that met requirements to depict how comprehensively each of the 25 analysed documents addresses the 9 core policy indicators across age target, location, WASH components, and stakeholder roles. Out of these 25 documents, only 4 achieved a perfect score of 9 (100%) fulfilment, including the Nairobi County Integrated Development Plan 2018–2022, the National pre-primary education policy, the Kenya Environmental Sanitation and Hygiene Policy, and the Policy Framework for Education. Conversely, the Footprints children’s home child protection policy scores the lowest, meeting only 1 out of 9 requirements (11%). Across all evaluated framework domains, the collective policy landscape satisfied approximately 63.5% of the total criteria, with a combined total of 143 successful integrations out of a maximum possible 225 across all documents.

### Policy context

Policy content, processes, and actors occur within a wider context by political, socio-economic, cultural and religious factors, in addition to specific aspects of the WASH system [38]. To understand this context, we reviewed each policy’s year of publication, focus, specific age group, and whether it addressed urban areas and informal settlements in its scope.

There were 25 policies related to early childhood development and childcare centres. Of the policies, 1 was at the county level while 24 were at the national level; 13 (53%) had clarity on under 5 years old children, 16 (64%) had guidelines on urban setting with 11 (44%) policies having guidelines on informal settlements.

Overall, the existing policies were not aligned, and many were inconsistent and did not adequately address WASH in childcare centre settings. This created overlapping roles resulting in procedural and substantive misunderstanding, as a proliferation of overlapping legislation inadvertently assigned duplicative regulatory roles to childcare centre service delivery stakeholders.

“*Many policies do not speak about informal settlements*,* but we are doing all we can to reach out to all institutions and education institutions everywhere in the count*y” (Policy Actor, KII-03).

“*Early childhood centres have access to WASH*,* including those in informal settlements. Although there are few that do not follow guidelines and we do all we can to ensure that there is compliance*” (Policy Actor, KII-06).

Study participants revealed that WASH initiatives in informal settlements often hinged on individual champions rather than institutional systems. For instance, when leadership changed, momentum was lost, and previous strategies were abandoned without evaluation or continuity. Socio-economic constraints were also emphasized. Parents and childcare centre providers cited poverty as a barrier to implementing WASH practices. For example, some families could not afford soap or clean water, and childcare centres lacked adequate funds for the service provision. Furthermore, cultural and behavioural norms played a dominant role in shaping hygiene habits. Participants noted that children’s hygiene behaviours were primarily shaped at home, yet many parents lacked the awareness or motivation to consistently reinforce positive WASH practices.

*“We just do not have the funds to maintain these services. It is hard to provide for the children when we cannot even afford basics like soap.” (Childcare provider*,* IDI-08).*

*“Hygiene starts at home*,* but with our financial struggles and inadequate knowledge*,* affording things like soap and clean water for my family is a real challenge.”* (Parent, IDI-03).

### Policy development process and actors

In the past, the policy development process in Kenya had limited public engagement. During our review, we investigated the extent of stakeholder participation in policy formulation. 16 out of the 25 policies (64%) indicated significant involvement from various actors. We deemed this involvement crucial for ensuring legitimacy during implementation. The stakeholders involved in the policy development phase included representatives from sectors such as government ministries, universities, research institutions, NGOs, community-based organizations (CBOs), faith-based organizations (FBOs), development partners, private sector entities, childcare providers, and parents. To develop policy frameworks and guidelines, several ministries and development partners formed a technical committee. This committee featured representatives from the health, education, labour, and social protection sectors. Study participants described a thorough process where stakeholders participated in various workshops and dialogues before finalising any policy.

“*The process of policy development is all inclusive*,* many actors are involved in policy development to enhance rightfulness in implementation”* (Policy Actor, KII-05).

There were notable dynamics of roles as portrayed by secondary data review compared to the data from primary data in Table 3.

**Table 3:**
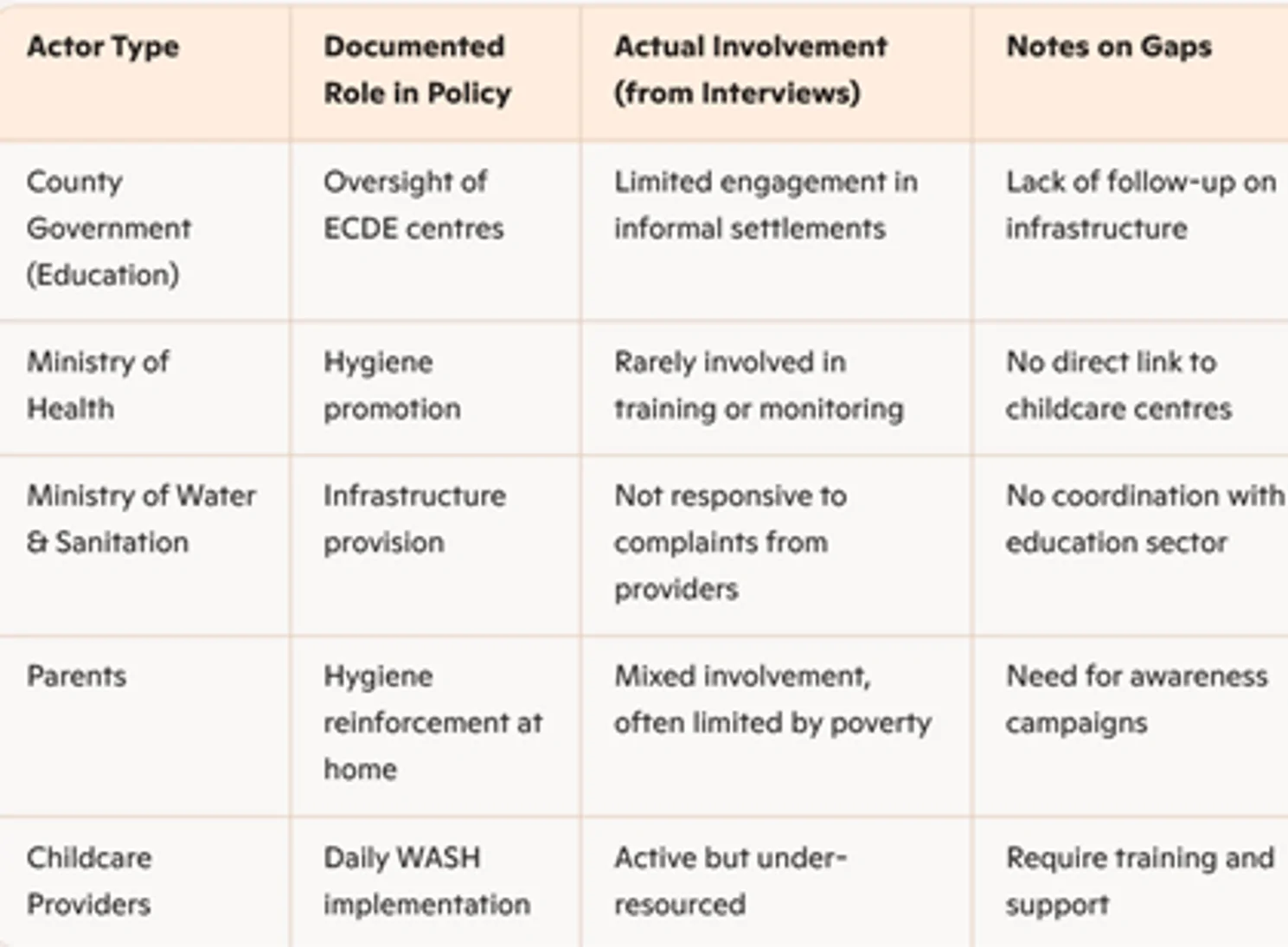
Actors involved versus practically engaged

While policies list a wide range of actors, government ministries, NGOs, parents and childcare providers, the primary data collection revealed that these actors were not actively involved. For instance, local administrations were described to have often ignored complaints from childcare providers about sanitation issues. Parents and childcare providers received minimum support, yet they were described as frontline day to day actors. Specifically, childcare providers lacked adequate capacity building initiatives, whereas parents faced economic and informational barriers. Despite being documented in policies, government departments responsible for water and sanitation rarely engaged with childcare centres. This contributed to poor service delivery and unmet needs. A lack or inadequacy in coordination among education, health, and water ministries was repeatedly a cited gap. Responsibilities were fragmented, and no single entity was accountable for WASH in childcare centres.

*“Our efforts are often fragmented across ministries*, edu*cation*,* health*,* and water*, l*eaving no single entity accountable for WASH in childcare centres”* (Policy Actor, KII-04).

### Policy implementation process and actors

We examined stakeholder involvement in implementing WASH policies. 20 out of the 25 policies reviewed (80%) clearly identified the actors responsible. These actors included parents, childcare providers, communities, both national and county governments, the private sector, civil society organizations (CSOs), CBOs, and FBOs. The review process showed how effective childcare programs were as the result of mutually dependent partnerships among these role players, who are responsible for the wellbeing and development of children in Kenya. Stakeholders and partners have diverse roles and responsibilities, including policy development, promotion and/or support of childcare centre services, provision of goods and services, and identification and promotion of peaceful coexistence initiatives.

With the multiplicity of players and roles, study participants described the existence of a collaborative framework for efficient coordination and management of childcare centre services and programs for children aged 0–8 years. The policy review process described parents as primary caregivers, with the community supporting and enhancing parents’ efforts in meeting the holistic needs of children. Crucially, interview data revealed that several actors with formally documented roles were rarely involved directly in policy development and implementation processes. This was highlighted as one reason for the ineffective policy responses, and poor service delivery. Many policies were reported to have been adopted from global policies, hence difficulty in implementation, monitoring and evaluation. Study participants also portrayed a lack of adequate knowledge on laws and policies, regulations and guidelines in the sector, depicting the need for awareness creation.

“*Policy implementation is the most challenging because sometimes people do not want to take up their responsibilities…But as always we offer capacity building on policy implementation*” (Policy Actor KII-02).

#### We present policy implementation gaps below:

a) *Lack of continuity of policy guideline due to change in leadership*.

Findings from the interviews showed that WASH interventions depended more on individual champions than on sustainable government structures.

*“As everyone wants to come up with innovative ideas*,* no one wants to evaluate*,* monitor and coordinate existing policies. So*,* all our funds are put into some new project*,* some innovation*,* hence always going back to square one. We should find out the strength and weaknesses of the various processes before starting a new project’* (Policy Actor, KII-07).

Participants reported that the reliance on individuals rather than robust structures and processes undermined long term planning and implementation. Study participants described that the weak structures and poorly established processes created a gap in the ownership of the policy structures. Additionally, evidence indicated that incoming leaders and entities frequently approached the same issue from a different perspective leading to the development of new policy guidelines while the existing ones were overlooked or abandoned. This discontinuity ultimately hindered the consistent implementation and limited the effectiveness of policy outcomes.

‘When leadership change due to political regime or other changes, *eventually the heads change*,* the view change …. Without evaluating the work*,* we have done we are asked to go’ (*Policy Actor, *KII-02).*

*b) Poor accountability of the local administration in the upkeep of WASH services in childcare centres*.

Childcare providers reported a distinct lack of sustained interest from local government regarding WASH infrastructure in childcare settings. They attributed this to inadequate or non-operational functions of National and County government such as water supply, sewage collection and disposal in childcare centres. Related to the 80% of policies with clearly defined actors, study participants highlighted that relevant departments addressing WASH service delivery in childcare centres lacked attention on the WASH and health needs of children in childcare centres.

*“The local government is more interested in older children than those in childcare centres. The local administration can install water filters and avail hand washing stations in childcare centres but it seems they are not interested and not responsive at all.’ (Childcare provider*,* IDI-06).*

Most childcare providers expressed dissatisfaction with the local administration for not accepting and considering complaints related to the water and sanitation infrastructure in childcare centres raised by Centre providers.

*‘We have written multiple reports with complaints to the local administration*,* but nobody hears our complaints … We complained about the lack of toilets’ (Childcare provider*,* IDI-08).*

Key informants suggested that a systematic approach could be imperative for successful administration of local government functions that enhance WASH and health needs of children in childcare centres.

*‘There is a need for all the local*,* and national ministries concerned to be accountable and responsible for strategies that enhance WASH and health needs of children in childcare centres’ (*Policy Actor, *KII-01).*

*c) Gaps in WASH planning and infrastructure*.

Key informants reported that several factors slowed down the implementation of WASH guidelines. These include: low WASH financing, and negative attitudes toward childcare centre among senior ministry staff. While the ministry implemented small portions of the policy, significant gaps remained. Childcare providers criticized the childcare centre curriculum for failing to teach hygiene concepts to children effectively. One provider argued that policies should clearly state all hygiene requirements, so teachers can instruct children accordingly.

‘… *All the things related to the hygiene of children should be explained and taught to us and should be clearly stated in the policies… so that we can teach the children on WASH related aspects accordingly’*. (Childcare providers, IDI-03)

Respondents noted that inadequate physical infrastructure and inattentiveness toward hygiene contributed to low hygiene literacy among the children. Teachers and parents perceived that childcare centre settings lacked the necessary resources to maintain a hygienic, germ-free environment for the children. These challenges included inadequate sanitation facilities, limited access to clean drinking water, absence of electricity and insufficient toilets.

*“There are few toilets in these childcare centres” (Parent*,* IDI-03)*.

Parents in particular highlighted non-functional sewage systems that risked the health of their children. One parent described how non-functioning water supplies forced children to rush home to use the toilet. Additionally, a childcare provider reported that drinking water from open channels contained visible dirt and germs.

*“My daughter has complained about the slippery toilets and non-functioning water supplies…in such a situation*,* some children like mine have to rush to their homes when they need to use a toilet” (Parent*,* IDI-02).*

‘*There is dirt in the drinking water that comes from the open channels. I am worried about the germs in the water. Children have to bring their water to childcare centres.*’ (Childcare provider, IDI-04).

*d) Interdepartmental coordination gaps and negligence*.

Over the last decade, the Kenyan government decentralized WASH service delivery to county authorities where different departments are responsible for coordination. Additionally, private providers and NGOs delivered WASH services widely across the country. This creates a complex network of institutions involved in WASH policy development and implementation. These fragmented structures negatively affected WASH guidelines in childcare centres. Actors often held duplicated roles, which left some essential WASH tasks unattended.

Key informants stressed the need for effective coordination in the water, sanitation, health, and education departments. Persistent poor coordination meant that the WASH needs of children remained unaddressed. While decentralization improved some processes, interview results highlighted significant limitations. One childcare provider noted that a lack of communication in these departments led to poor sanitation practices. Another provider argued that better contact between health and education officials would benefit both children and parents.

‘*There is a lack of effective communication among WASH*,* health and education departments that leads to poor sanitation practices in childcare centres.’ (Childcare provider IDI-06*).

‘*There should be communication in government departments … It will be beneficial to the children and the parents … For example*,* health department people should get in contact with the water*,* sanitation*,* hygiene and education departments… It will lead to improvement*.’ (Childcare provider, IDI-08).

Policy guidelines assigned specific roles to parents in WASH provision. Providers pointed out that parental negligence negatively impacted children’s hygiene habits. However, these could be attributed to factors such as poverty and existing household behaviours.

*‘Parents have a great role in implementing some WASH guidelines…for example in ensuring children are clean when they arrive at the centre in the morning. Some parents are careless towards their children’s WASH habits…Parents tend to withdraw their children from the centre if children were asked for anything beyond studies. Poverty was also an underlying factor affecting children’s WASH behaviour*,* as some parents cannot afford anything that necessitates personal hygiene”* (Childcare provider, IDI-05).

Childcare providers believed parents were key to behaviour change, yet many parents lacked knowledge of good hygienic practices. These unhygienic practices often passed from parents to children. A lack of parental motivation further amplified the problem. Providers noted that children copy their parents, who often do not wash their own hands. Conversely, parents reported that childcare providers neglected the hygiene needs of children once they arrived at the centre.

‘*A child first learns from home. If parents never portray or educate children on good hygiene*,* then children end up not keeping hygiene and maintain good behaviours.’* (Childcare provider, IDI-07).

*“Some parents lack awareness of hygiene and are not motivated to observe hygienic behaviours. They do not even wash hands and children ends up copying the same habits” (Childcare providers*,* IDI-02)*.

Some parents complained that staff failed to provide water for handwashing during the day.

‘*We do our part sometimes as parents*,* but childcare providers neglect our children while they are in childcare centres and so children do not access water for handwashing at sometimes*” (Parent, IDI-5).

## Discussion of findings

This study addresses critical literature gaps by examining the WASH policy landscape within childcare centres in Nairobi’s informal settlements. Using the Walt and Gilson’s Policy Triangle Framework, the analysis revealed systemic deficiencies across the four dimensions of context, process, content, and actors which collectively compromised WASH service delivery. These findings are particularly important given the existing evidence that the integration of quality WASH standards in childcare settings enhance children’s health [20–22] and improve their wellbeing [39]. Further, it has potential to establish positive WASH-related attitudes and behaviours for sustainable management of WASH services and practices, among children before school-entry age enabling them to act as future champions [15, 22].

The identified policy gaps illustrated the need for an understanding of the policy gaps to promote approaches that ensure safety, equity, accessibility, availability and affordability of WASH services for settings where children live, study, play, rest and seek care. In Kenya, the WASH policy landscape for childcare centres is characterized by multiple policy actors [8]. While these actors are actively involved in policy formulation, implementation remains constrained by several interrelated factors inconsistent leadership, poor accountability from local administrations, inadequate training and infrastructure, and a lack of interdepartmental coordination. Additionally, gaps in knowledge and practices among parents and childcare providers further compound these challenges. Collectively, these structural deficiencies paralyze WASH service delivery within informal settlement childcare centres. Evidence from a study in Malawi demonstrated that the effective implementation of a policy guidelines on WASH can lead to a significant rise in childcare centre cleanliness (*p* < 0.01) scores among children, and reduced absenteeism [40, 41].

Application of the Policy Triangle Framework revealed a critical disconnect between policy formulation and practical execution [25, 42]. Unlike traditional policy analyses that focus primarily on content, this framework highlights the dynamic interplay in policy content, implementation context, processes, and actors. The findings illustrate how the different components of the policy triangle [26] are interconnected with implementation outcomes shaped not only by policy design but also by institutional capacity, actor engagement and contextual realities. This interconnection explains why well-developed policies often fail to translate into effective actions. With respect to actors involved in policy development and implementation, the study found out that decisions made, and the course of action advocated are dependent on individual actors than on sustainable structures. This undermines the continuity and sustainability of the process. When policies are not implemented as intended, it can undermine the motivation of those who are meant to carry out the strategy [43].

The importance of continuity in implementing policy guidelines is supported by evidence from policy reforms in other contexts, where a change of leadership in the government triggers shift in focus and loss of momentum in policy implementation [44]. In this study, continuity is linked to ownership suggesting that greater involvement of actors with responsibilities for implementation in policy development could encourage local ownership and allow better assessment of policy feasibility. Involvement of a range of actors can strengthen the policy processes by bringing new perspectives [38] and encouraging complementarity of functions. However, findings from this study also highlight coordination challenges rooted in the context in which WASH practices and interventions in childcare centres were implemented. The fragmentation of responsibilities across ministries, specifically education, health, and water mean that no single entity is held accountable for overseeing and ensuring the provision of services. This lack of a coordinated structure creates an environment where initiatives are easily stalled and sustainable progress is difficult to achieve.

In relation to policy development and context, the study revealed that improving WASH services in childcare centres is dependent on multiple contextual factors operating across policy development and implementation. Similar results can be found in a comparative study of policy development processes in Sub-Saharan Africa where coordination that span across different sectors and departments during the development phase was found to be a key factor in successful policy implementation [45–47]. Additionally, the findings suggest that capacity to plan is key for success of policy development and ultimately implementation. Potter and Brough [46] noted a lack of capacity in the Indian health and family welfare sector in terms of structures and processes that allow managers and other leaders to contribute to policy development [46]. A notable finding of this study is the divergence between policy as articulated in formal documents and policy as experienced by stakeholders on the ground. Although numerous policies exist, they often fail to provide specific guidelines for WASH in childcare centres within informal settlements leading delivery challenges. The challenges outlined in implementation are strongly corroborated by existing literature on WASH service delivery in Nairobi’s informal settlements [23, 33].

Despite Kenya’s adoption of frameworks like the Integrated Early Childhood Development Policy and the Early Childhood Education Act, WASH services in childcare centres remain inadequate due to fragmented implementation and weak accountability mechanisms [32, 47]. Studies reveal that while policies enumerate multiple stakeholders, departments such as water and sanitation often lack direct engagement with childcare centres, resulting in poor service delivery [31]. Informal social accountability mechanisms have emerged as a response to the absence of formal structures, with parents and childcare providers, key frontline actors bearing the burden of service provision despite limited resources and support [31, 47]. These findings affirm that policy-practice gaps, leadership instability, and interdepartmental disconnects critically undermine WASH outcomes in these vulnerable settings.

The persistent inadequacies in WASH service delivery within Nairobi’s informal childcare centres are not merely symptomatic of localized policy gaps, but are deeply entrenched in the structural challenges of Kenya’s devolved governance system [30]. While decentralization aimed to enhance local service delivery, the diffusion of WASH responsibilities across the Ministries of Health, Education, and Water and Sanitation has inadvertently generated overlapping institutional mandates, resulting in a profound breakdown in systemic accountability where no single entity takes ownership [24, 39]. This fragmented regulatory architecture is further exacerbated by the political economy of urban informality, where poverty forces under-resourced childcare providers and parents to navigate precarious, informal networks to meet basic hygiene and sanitation needs [31, 47]. By situating these realities within the broader sub-Saharan African public health and urban planning literature, our findings underscore that achieving sustainable impact requires moving beyond descriptive policy formulation to critically resolve the deep-seated political and institutional disconnections that chronically paralyze WASH governance in marginalized urban spaces.

Kenya’s policy landscape was depicted to be highly siloed, causing unequal integration in early childhood development, urban poverty, and sanitation frameworks. Devolved frameworks like the Nairobi County Integrated Development Plan successfully achieve perfect alignment by directly connecting institutional childcare needs to the realities of informal settlements. Specialized child protection and social safety policies score poorly because they prioritize legal rights while completely omitting essential environmental determinants of safety, such as clean water and functional hygiene infrastructure. Globally, these findings strongly mirror the implementation-aspiration gap and the multi-sectoral policy paradox frequently cited in public health and development literature [45, 46]. While international frameworks like the World Health Organization’s Nurturing Care Framework champion simultaneous interventions in health, learning, and sanitation, academic research highlights that rigid ministerial boundaries (such as Education vs. Water) often prevent these ideals from being co-authored into law [47]. Furthermore, literature on urban health equity emphasizes that national frameworks frequently use sweeping, universal language that fails to account for the unique legal and infrastructural challenges of informal settlements [44, 45]. While Kenya has successfully authored highly comprehensive anchor policies, the immediate strategic priority is not to create new legislation, but to use these top-tier frameworks to modernize and cross-pollinate the country’s isolated, single-sector policies.

## Strengths and limitations of the study

To the best of our knowledge, this is the first study that yielded evidence on gaps in water, sanitation and hygiene policies by reviewing the WASH policy guidelines in childcare centres in existing policies in Kenya. Although the study involved only key and influential stakeholders in Nairobi’s informal settlements, WASH policy gaps identified from our study may be applicable to childcare centres in other contexts.

## Conclusion

The study demonstrated that there is a disconnect between WASH policy formulation and practical execution in childcare centres within Nairobi’s informal settlements. The policy landscape is functionally compromised by a mismatch between policy design and implementation. Most policy documents lacked the necessary specificity required to address the realities of children under five in informal urban settlements. This contextual misalignment, compounded by persistent poverty-related barriers, renders service delivery efforts largely non-operational and unsustainable at the point of need.

Successful translation of WASH policy into practice requires a shift in focus beyond policy content to include policy process and actor responsibility. Previous policy review processes have focused more on policy content overlooking the critical interplay in the context, process and actors. This study addresses that gap by providing a comprehensive analysis of WASH policy landscape in relation to the childcare centres in informal settlements. Developing and implementing policy guidelines requires involvement from relevant stakeholders, clear delineation of responsibilities, and political commitment.

We also conclude that optimizing these environments demands a governance paradigm shift that moves beyond simple policy formulation to the establishment of a cohesive, politically supported, and rigorously monitored architecture capable of enforcing standardized WASH provisions across all relevant ministerial domains. Addressing these systemic gaps is essential for translating policy intent into tangible public health benefits and offers broader implications for improving policy implementation in other sectors and levels of education operating in decentralized, resource-constrained urban environments.

## Recommendations for policy and practice

To address the current WASH policy misalignment, the government and relevant actors should:

i. Ring-fence WASH financing mechanism targeting informal childcare centres.
ii. Co-design concrete institutional tracking tools for improved monitoring and accountability.
iii. Integrate context-specific protocols and planning into policy iterations that directly target the unique infrastructure and safety needs of informal urban settlements and children under five.
iv. Shift the administrative focus away from merely formulating policy documents toward building a cohesive, cross-sectoral architecture that is continuously monitored to ensure high-level goals translate into sustainable, point-of-need service delivery.
v. Move beyond generic policy language to secure strong political support for a governance paradigm that enforces standardized, inclusive WASH provisions across all relevant ministries.
vi. Co-design a unified WASH checklist for informal childcare centres that bridges the current gap between Ministry of Water, Sanitation and Irrigation, Ministry of Health and Ministry of Education mandates.
vii. Formalize Community-led monitoring models like Childcare WASH Committees involving parents and providers to ensure sustained maintenance of facilities, which can be integrated into the existing community health promoters’ framework.

To achieve sustainable improvements in WASH service delivery and child health outcomes, a concerted effort is required to: [1] establish clear legislative backing and delineation of responsibilities among all relevant actors; [2] foster stronger political commitment and institutional ownership to ensure strategy continuity; and [3] enhance the capacity and coordination of WASH systems within Nairobi’s informal settlements. Research on the WASH policy landscape in childcare settings has broader implications for education policies. Other levels of education could consider these findings within their own contexts.

## Supplementary Information

Supplementary Material 1.

Supplementary Material 2.

## Data Availability

The data presented in this study are available on request from the corresponding author. The data are not publicly available because other manuscripts are still being written using the same data.

## References

[1] United Nations Children’s Fund (UNICEF) and World Health Organization. 2018. Drinking Water, Sanitation and Hygiene in Schools: Global baseline report 2018. UNICEF. New York, USA; 2018:1–84 p.

[2] Jiménez A, Livsey J, Åhlén I, Scharp C, Takane M Global assessment of accountability in water and sanitation services using GLAAS data. Water Altern. 2018;11(2):238–59. https://www.water-alternatives.org/index.php/alldoc/articles/vol11/v11issue2/435-a11-2-2/file.

[3] Dooley MS, Water T. Sanitation and Hygiene (WASH) in Schools. 1st ed. UNICEF, editor. New York, USA: UNICEF; 2012. pp. 1–56.

[4] Kim J, Laituri M. An examination of water, sanitation, and hygiene accessibility and opportunity in urban informal settlements during the COVID-19 pandemic: Evidence from Nairobi, Kenya. Sci Total Environ. 2022. 10.1016/j.scitotenv.2022.153398. PMC8799381 35092785

[5] UNICEF W. Progress on drinking water, sanitation and hygiene in schools: 2000–2021 data update. New York, USA; 2022.

[6] Ngwacho AG. COVID-19 Pandemic Impact on Kenyan Education Sector: Learner Challenges COVID-19 Pandemic Impact on Kenyan Education Sector. Learner Challenges and Mitigations; 2020.

[7] Worrell CM, Wiegand RE, Davis SM, Odero KO, Blackstock A, Cuéllar VM, et al Hygiene-related risk factors for soil- transmitted helminth infection in urban school- and preschool-Aged children in Kibera, Nairobi. PLoS ONE. 2016;11(3). 10.1371/journal.pone.0150744. PMC4780697 26950552

[8] Wagner JT, Wagner JT, Pramling I WASH from the START: Water, Sanitation and Hygiene Education in Preschool. Int J Early Child. 2019;51(1):5–21. Available from: 10.1007/s13158-019-00236-5.

[9] Tata J. Early Childhood Care and Education in Kenya. Int J Early Child. 2012;15:2001–2. 10.1080/00094056.2004.10522230 2012; 18(4) 191–197.

[10] Kibuchi E, Barua P, Chumo I, Teixeira N, Filha DS, Howard PP- et al Effects of social determinants on children’ s health in informal settlements in Bangladesh and Kenya through an intersectionality lens: a study protocol. 2022;1–7. 10.1136/bmjopen-2021-056494. PMC9171224 35667712

[11] Clark S, De Almada M, Kabiru CW, Muthuri S, Wanjohi M Balancing paid work and childcare in a slum of Nairobi, Kenya: the case for centre-based childcare. J Fam Stud. 2021;27(1):93–111. Available from: 10.1080/13229400.2018.1511451.

[12] Nganga L. Early childhood education programs in Kenya: challenges and solutions. Early Years. 2016;29(3):227–36. 10.1080/09575140902984400.

[13] Stella Muthuri D, Doughman C, Mitchell. and AD. Exploring the challenges in combining work and care for young children: Use of PhotoVoice with mothers from an urban Nairobi slum. Vol. 1. 2016.

[14] Bassett L, Dc W. The Early Childhood Development Action Network: a global network to strengthen support for young children. 2017;93–7.

[15] Mcmichael C, Water. Sanitation and Hygiene (WASH) in Schools in Low-Income Countries: A Review of Evidence of Impact. Int J Environ Res Public Heal Rev. 2019;16(359):1–21. 10.3390/ijerph16030359 PMC6388361 30696023

[16] Ngigi S. Assessment of the Early Childhood Development Policy Implementation in Kenya, Case Study of Ruiru District Catholic University of Eastern Africa. J Educ Soc Policy. 2015;2(1):78–89.

[17] Mansour G, Oyaya C, Owor M. Situation analysis of the urban sanitation sector in Kenya. Urban Sanit Res Initiat Ghana [Internet]. 2017;(July):27. Available from: http://www.wsup.com/resource/situation-analysis-of-the-urban-sanitation-sector-in-bangladesh/?platform=hootsuite

[18] Ezeh A, Oyebode O, Satterthwaite D, Chen Y, Ndugwa R, Sartori J, Mberu B, et al. The history, geography, and sociology of slums and the health problems of people who live in slums. Lancet. 2016;389(10068):547–58. 10.1016/s0140-6736(16)31650-6. 27760703

[19] Thorne G, Carvajal A, Kim W. Early childhood development in urban informal settlements and slums. 1st ed. editor. Geneva, Switzerland: Save the Children; 2021. pp. 1–66.

[20] IRC 2007. Towards Effective Programming for WASH in Schools; 2007.

[21] UNICEF, Baby WASH, Programming. UNICEF Eastern and Southern Africa Learning Note 2020; 2020.

[22] Chumo I, Kabaria C, Phillips-Howard PA, Ozano Kim, Elsey H, Mberu B. Co-creation and self-evaluation: An accountability mechanism process in water, sanitation and hygiene services delivery in childcare centres in Nairobi’s informal settlements. Front Public Health 2023 Jan 12:101035284. 10.3389/fpubh.2022.1035284 PMC9877527 36711348

[23] Chumo I, Mberu B, Wainaina C, Murigi W, Sumba L, Kabaria C. Sanitation services for the urban poor: A social capital approach to sanitation challenges in informal settlements. PLOS Water [Internet].2023;2(12):e0000086. Available from: 10.1371/journal.pwat.0000086

[24] Brien GLO, Sinnott S, Walshe V, Mulcahy M, Byrne S. Health policy triangle framework: Narrative review of the recent literature. Heal Policy OPEN [Internet]. 2020;1:100016. Available from: 10.1016/j.hpopen.2020.100016 PMC10297800 37383316

[25] Gilson L, Raphaely N. The terrain of health policy analysis in low and middle-income countries: a review of published literature 1994–2007. 2008;294–307. 10.1093/heapol/czn019 PMC2515407 18650209

[26] Walt G, Gilson L. Reforming the health sector in developing countries: The central role of policy analysis. Health Policy Plan. 1994;9(4):353–70. 10.1093/heapol/9.4.353. 10139469

[27] Abboah-Offei M, Amboka P, Nampijja M, Owino GE, Okelo K, Kitsao-Wekulo P, et al. Improving early childhood development in the context of the nurturing care framework in Kenya: A policy review and qualitative exploration of emerging issues with policy makers. Front Public Heal. 2022;10(1):1–25. 10.3389/fpubh.2022.1016156. PMC9551223 36238244

[28] APHRC. (MWS&I) and Ministry of water sanitation irrigation. Development of the national sanitation management policy (NSMP) in Kenya. 2022.

[29] Beguy D, Elung P, Mberu B, Oduor C, Wamukoya M, Nganyi B, et al. HDSS Profile: The Nairobi Urban Health and Demographic Surveillance System (NUHDSS). Int J Epidemiol. 2015;1–10. 10.1093/ije/dyu251. 25596586

[30] Emina J, Beguy D, Zulu EM, Ezeh AC, Muindi K, Elung P et al. Monitoring of Health and Demographic Outcomes in Poor Urban Settlements: Evidence from the Nairobi Urban Health and Demographic Surveillance System. 2011;88:200–18. 10.1007/s11524-011-9594-1 PMC3132229 21713553

[31] Chumo I, Kabaria C, Phillips-Howard PA, Simiyu S, Elsey H, Mberu B. Mapping social accountability actors and networks and their roles in water, sanitation and hygiene (WASH) in childcare centres within Nairobi’s informal settlements: A governance diaries approach. PLoS ONE. 2022;17(11 November):1–21. 10.1371/journal.pone.0275491. PMC9665391 36378662

[32] Chumo I, Kabaria C, Phillips-Howard PA, Gitau H, Elsey H, Mberu B. Beyond a facility: WASH service levels and informal social accountability in childcare centres in informal settlements. J Water Sanit Hyg Dev. 2024;00(0):1–12. 10.2166/washdev.2024.177.

[33] Chumo I, Mberu B, Kabaria C How informal water markets serve the urban population: Dynamics of invisible hand in Nairobi’s water sector. PLOS Water. 2025;4:1–17. Available from: 10.1371/journal.pwat.0000379.

[34] Chumo I, Kabaria C, Shankland A, Igonya E, Mberu B Complementarity of formal and informal actors and their networks in support of vulnerable populations in informal settlements: Governance diaries. Front Public Heal. 2023;10. 10.3389/fpubh.2022.1043602. PMC9911518 36777785

[35] Chumo I, Kabaria C, Oduor C, Amondi C, Njeri A, Mberu B. Community advisory committee as a facilitator of health and wellbeing: A qualitative study in informal settlements in Nairobi, Kenya. Front public Heal. 2022;10(1047):1–12. 10.3389/fpubh.2022.1047133. PMC9868729 36699924

[36] Liu X, Burnett D. Insider-outsider: Methodological reflections on collaborative intercultural research. Humanit Soc Sci Commun. 2022;9(1):1–8. 10.1057/s41599-022-01336-9.

[37] Nicola Gale G, Heath. Elaine Cameron SR and SR. Using the framework method for the analysis of qualitative data in multi-disciplinary health research. BMC Med Res Methodol. 2013;13(117):1–8. 10.1186/1471-2288-13-117. PMC3848812 24047204

[38] Green A, Gerein N, Mirzoev T, Bird P, Pearson S, Anh LV et al Health policy processes in maternal health: A comparison of Vietnam, India and China. Health Policy (New York). 2011;100(2–3):167–73. Available from: 10.1016/j.healthpol.2010.11.016. 21194780

[39] Kenya R. of. Kenya’s Constitution of 2010. 2010 p. 1–308.

[40] Mwapasa T, Chidziwisano K, Lally D, Morse T Hygiene in early childhood development centres in low-income areas of Blantyre, Malawi. Int J Environ Health Res [Internet]. 2022;00(00):1–17. Available from: 10.1080/09603123.2022.2048802. 35272551

[41] Manda MAZ. Water and sanitation in urban Malawi: Can the Millennium Development Goals be met? A study of informal settlements in three cities; 2009.

[42] Sheikh K, Saligram PS, Hort K What explains regulatory failure? Analysing the architecture of health care regulation in two Indian states. 2013;1–17. Health Policy Plan. 2015;30(1):39–55. 10.1093/heapol/czt095. 24342742

[43] Paul Cairney. Understanding Public Policy: Theories and Issues. Public Adm. 2013;91(3):782–93.

[44] Khan MM, Van den Heuvel W. The impact of political context upon the health policy process in Pakistan. Public Health. 2007;121(4):278–86. 10.1016/j.puhe.2006.09.015. 17217971

[45] Walt G, Shiffman J, Schneider H, Murray SF, Brugha R. ‘Doing’ health policy analysis: methodological and conceptual reflections and challenges. 2008;(2000):308–17. 10.1093/heapol/czn024. PMC2515406 18701552

[46] Potter C, Brough R. Systemic capacity building: A hierarchy of needs. Health Policy Plan. 2004;19(5):336–45. 10.1093/heapol/czh038. PMid:15310668. 15310668

[47] Chumo I, Kabaria C, Muindi K, Elsey H, Phillips-howard PA, Mberu B Informal social accountability mechanisms for water sanitation and hygiene (WASH) in childcare centres in Nairobi City County’s informal settlements. Urban Gov. 2022;1(1):1–11. Available from: 10.1016/j.ugj.2022.07.001.

