# Supplementary material for: WASH policy landscape: exploring context, process, content, and actors in childcare centres in Nairobi’s informal settlements
Source: BMC Public Health. 2026 Jul 24;26:3019. doi: 10.1186/s12889-026-28653-x (PMC13685175; doi:10.1186/s12889-026-28653-x)
Supplement: Supplementary file 1 [file 12889_2026_28653_MOESM1_ESM.pdf]

SPRINGER NATURE

Change of authorship request form - Journals (pre-acceptance)

Important information. Please read.

- This form should be used by authors to request any change in authorship (adding/deleting authors) including changes in corresponding authors. This form should not be used for name changes. Please fully complete all sections. Use black ink and block capitals and provide each author's full name with the given name first followed by the family name.

- By signing this declaration, all authors guarantee that the order of the authors are in accordance with their scientific contribution, if applicable as different conventions apply per discipline, and that only authors have been added who made a meaningful contribution to the work.

- Please note, in author collaborations where there is formal agreement for representing the collaboration, it is sufficient for the representative or legal guarantor (usually the corresponding author) to complete and sign the Authorship Change Form on behalf of all authors, **next to the added/removed author(s)**. **(Complete Section 3, followed by Section 6.)**

  In author collaborations where there is no formal agreement for representing the collaboration and **there are more than 10 authors**, one may sign for all, provided the signer appends correspondence that attests that each of the authors have agreed to the change **and the added/removed authors sign the form**. **(Complete Section 3, followed by Section 6.)**

- Please note, we cannot investigate or mediate any authorship disputes. If you are unable to obtain agreement from all authors (including those who you wish to be removed) you must refer the matter to your institution(s) for investigation. Please inform us if you need to do this.

- If you are not able to return a fully completed form within **30 days** of the date that it was sent to the author requesting the change, we may have to withdraw your manuscript. We cannot publish manuscripts where authorship has not been agreed by all authors (including those who have been removed).

- Incomplete forms will be rejected.

- Please return/upload this form, fully completed, to the Journals Editorial Office. The Journal and/or Publisher will consider the information you have provided to decide whether to approve the proposed change in authorship. We may decide to contact your institution for more information or undertake a further investigation, if appropriate, before making a final decision.

SPRINGER NATURE

Change of authorship request form - Journals (pre-acceptance)

Section 1: Please provide the current title of manuscript

Manuscript ID no.: bc1d2943-d875-4ec4-80e3-0fe351996377

Title: WASH policy landscape: Exploring context, process, content, and actors in childcare centres in Nairobi's informal settlements

Section 2: Please provide the previous authorship, in the order shown on the manuscript before the changes were introduced. Please indicate the corresponding author by adding (CA) behind the name.

	First name(s)	Family name	ORCID or SCOPUS id, if available
1st author	Ivy	Chumo	https://orcid.org/0000-0003-1235-719X
2nd author	Caroline	Kabaria	https://orcid.org/0000-0002-5641-5243
3rd author	Sarah	Munyao Ndonye	n/a
4th author	Helen	Elsey	https://orcid.org/0000-0003-4724-0581
5th author	Penelope	A Phillips-Howard	https://orcid.org/0000-0003-1018-116X
6th author	Blessing	Mberu	https://orcid.org/0000-0002-6188-196X
7th author			
8th author			
9th author			
10th author			

Please use an additional sheet if there are more than 10 authors.

Section 3: Please provide a justification for change. Please use this section to explain your reasons for changing the authorship of your manuscript, e.g. what necessitated the change in authorship? Please refer to the (journal) policy pages for more information about authorship. Please explain why omitted authors were not originally included and/or why authors were removed on the submitted manuscript.

SPRINGER NATURE ---

Springer Nature is one of the world's leading global research, educational and professional publishers, created in May 2015 through the combination of Nature Publishing Group, Palgrave Macmillan, Macmillan Education and Springer Science+Business Media.

SPRINGER NATURE

Change of authorship request form - Journals (pre-acceptance)

We have removed Sarah Munyao Ndonye from the author list and acknowledge her contribution in the Acknowledgements section instead. She contributed in the editorials of the manuscript

Section 4: Proposed new authorship. Please provide your new authorship list in the order you would like it to appear on the manuscript. Please indicate the corresponding author by adding (CA) behind the name. If the Corresponding Author has changed, please indicate the reason under section 3.

	First name(s)	Family name (this name will appear in full on the final publication and will be searchable in various abstract and indexing databases)	Affiliated institute	E-mail address
1st author	Ivy	Chumo	African Population and Health Research Centre (APHRC), Nairobi, Kenya	ivychumo@gmail.com
2nd author	Caroline	Kabaria	African Population and Health Research Centre (APHRC), Nairobi, Kenya	ckaberia@aphrc.org,
3rd author	Helen	Elsey	University of York, and Hull York Medical School, York, UK	hrlsey@uoy.uk.ac,
4th author	Penelope	A Phillips-Howard	[3] Liverpool School of Tropical Medicine, Department of Clinical Sciences, Liverpool, UK	pphiliphs@lstmed.uk.ac,
5th author	Blessing	Mberu	African Population and Health Research Centre (APHRC), Nairobi, Kenya; and Demography and Population Studies, University of Witwatersrand, Johannesburg, South Africa	bmbe@aphrc.org

SPRINGER NATURE ---

Springer Nature is one of the world's leading global research, educational and professional publishers, created in May 2015 through the combination of Nature Publishing Group, Palgrave Macmillan, Macmillan Education and Springer Science+Business Media.

Change of authorship request form - Journals (pre-acceptance)

7th author				
8th author				
9th author				
10th author				

Please use an additional sheet if there are more than 10 authors.

Section 5: Author contribution, Acknowledgement and Disclosures. Please use this section to provide a new disclosure statement and, if appropriate, acknowledge any contributors who have been removed as authors and ensure you state what contribution any new authors made (if applicable per the journal or book (series) policy). Please ensure these are updated in your manuscript - after approval of the change(s) - as our production department will not transfer the information in this form to your manuscript.

New acknowledgements:

We acknowledge all study participants, study community members, WASH policy review team at APHRC, Childcare policy review team, Research team, APHRC staff and ARISE consortium members for their guidance and input during this project.

New Disclosures (financial and non-financial interests, funding):

GCRF Accountability funded the project activities through UKRI Collective Fund award with award reference ES/S00811X/1.

New Author Contributions statement (if applicable per the journal policy):

Reviews: Sarah Munyao Ndonye contributed to language modification in the manuscript.

State 'Not applicable' if there are no new authors.

Section 6: Declaration of agreement. *All* authors, unchanged, new and removed *must* sign this declaration.
(NB: Please print the form, (docu)-sign and return/upload a scanned copy. Please note that signatures that have been inserted as an image file are acceptable as long as it is handwritten. Typed names in the signature box are unacceptable.) *** Please delete as appropriate. Delete all of the bold if you were on the original authorship list and are remaining as an author.**

	First name	Family name		Signature	Date
1st author	Ivy	Chumo	I agree to the proposed new authorship shown in section 4 /**and the addition/removal*of my name to the authorship list** /and the proposed change in corresponding author		10/07/2026
2nd author	Caroline	Kabaria	I agree to the proposed new authorship shown in section 4 /**and the addition/removal*of my name to the authorship list** /and the proposed change in corresponding author		11/07/2026
3rd author	Helen	Elsey	I agree to the proposed new authorship shown in section 4 /**and the addition/removal*of my name to the authorship list** /and the proposed change in corresponding author		11/07/2026

SPRINGER NATURE ---

Springer Nature is one of the world's leading global research, educational and professional publishers, created in May 2015 through the combination of Nature Publishing Group, Palgrave Macmillan, Macmillan Education and Springer Science+Business Media.

SPRINGER NATURE

Change of authorship request form - Journals (pre-acceptance)

4th authors	Sarah	Munyao Ndonye	I agree to the proposed new authorship shown in section 4 **/and the addition/removal*of my name to the authorship list** /and the proposed change in corresponding author	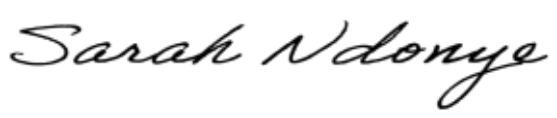	12/07/2026
5th author	Penelope	A Phillips-Howard	I agree to the proposed new authorship shown in section 4 **/and the addition/removal*of my name to the authorship list** /and the proposed change in corresponding author	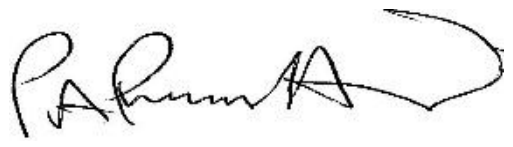	13/07/2026
6th author	Blessing	Mberu	I agree to the proposed new authorship shown in section 4 **/and the addition/removal*of my name to the authorship list** /and the proposed change in corresponding author	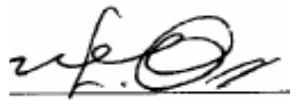	13/07/2026
7th author			I agree to the proposed new authorship shown in section 4 **/and the addition/removal*of my name to the authorship list** /and the proposed change in corresponding author		

	First name	Family name		Signature	Date
8th author			I agree to the proposed new authorship shown in section 4 **/and the addition/removal*of my name to the authorship list** /and the proposed change in corresponding author		
9th author			I agree to the proposed new authorship shown in section 4 **/and the addition/removal*of my name to the authorship list** /and the proposed change in corresponding author		
10th author			I agree to the proposed new authorship shown in section 4 **/and the addition/removal*of my name to the authorship list** /and the proposed change in corresponding author		

SPRINGER NATURE ---

Springer Nature is one of the world's leading global research, educational and professional publishers, created in May 2015 through the combination of Nature Publishing Group, Palgrave Macmillan, Macmillan Education and Springer Science+Business Media.

Change of authorship request form - Journals (pre-acceptance)

Please use an additional sheet if there are more than 10 authors.

In case of author collaborations with formal agreement:

	Name of consortium/consortia	First name	Family name		Signature	Date
Representative/legal guarantor				I agree to the proposed new authorship shown in section 4 /**and the addition/removal*of my name to the authorship list** /and the proposed change in corresponding author		

Both added/removed authors should complete the information in the first table under Section 6.

---- End of form ----
